# Genetic Diversity and Demographic History of Globe Skimmers (Odonata: Libellulidae) in China Based on Microsatellite and Mitochondrial DNA Markers

**DOI:** 10.1038/s41598-019-45123-0

**Published:** 2019-06-13

**Authors:** Ling-zhen Cao, Kong-ming Wu

**Affiliations:** 1 Institute of Plant Protection, Chinese Academy of Agricultural Sciences, Beijing, 100193 P.R. China; 2 College of Life Science, Jiangxi Normal University, Nanchang, Jiangxi 330022 P.R. China

**Keywords:** Molecular biology, Ecology

## Abstract

To analyze genetic characters of migratory dragonflies, we used 10 microsatellite markers and a partial sequence of the mitochondrial gene *Cytb* to investigate genetic diversity and demographic history among 19 populations of *P. flavescens* in eastern the monsoon region of China. In a Bayesian clustering analysis of the microsatellite data, three distinct clades were present, and each population consisted of a mixture of individuals from the three clusters. An AMOVA of the data from both the microsatellite loci and *Cytb* revealed that genetic variation was mainly within each population. For the 543 individuals from the 19 regions, 77 unique haplotypes were obtained by DnaSP 4.0, and a median-joining network showed no obvious geographical pattern and displayed high gene flow and minimal population genetic structure among the 19 populations. According to a Mantel test, there was no significant association between genetic distance and geographic distribution and no isolation by distance. Mismatch distribution and neutrality tests showed no demographic expansion for the 19 populations. Microsatellite and mitochondrial DNA data suggested there was high gene flow and low differentiation among the populations. These results will help provide valuable information to study the migratory route of insects, especially important agricultural pests.

## Introduction

Animal migration is one of the most remarkable phenomena in nature, in pursuit of improved safety, increased foraging opportunities, and higher reproductive output^1,2^. When it comes to long distance migrations, some animals, such as birds and whales, may be the most commonly thought of^3^. Yet, some dragonflies are as capable of long- distance travel. Insects in the order Odonata (phylum Arthropoda, class Insecta, subclass Pterygota) are the most ancient invertebrates capable of flight and very diverse, with approximately 6000 species worldwide^4–6^. Mass migration of dragonflies has long been known and there are about 25–50 migratory species^6,7^. *Pantala flavescens* is well suited for phylogeographic studies due to its extensive migratory ranges, which spans mountain ranges, continents and oceans.

*Pantala flavescens* (Fabricius, 1798) may be the most common dragonfly in the world because it is very adaptable to different habitats and long-distance dispersal capability. It occurs in temperate and tropics regions, from lowland to montane, typically in coastal and open areas that span thousands of kilometers^8–11^. Isotopic evidence also suggests that its multigenerational journey may total over 18,000 km, with single individuals traveling over 6,000 km during the transoceanic trek from northern India to east Africa^12^.

Such migratory behavior of odonate insects could homogenize genetic differentiation among populations by the exchange of individuals and genes among populations that are separated by large distances and thus impact the population structure of that species. For example, haplotype diversity in populations of *Anax junius* (Drury 1773), the common green darner, is relatively high in the absence of any obvious phylogeographic pattern^13^. *Libellula quadrimaculata* (Linnaeus, 1758), the four-spotted chaser or skimmer, showed high haplotype interconnection among samples collected within Asia, Europe and North America^14^.

Previous research on *P. flavescens* using randomly amplified polymorphic DNA has also shown that genetic diversity is low and gene flow is high among five geographically isolated populations within India^15^. Based on sequence data using cytochrome oxidase 1 (*CO1*), high rates of gene flow are present among all studied geographic regions and genes are shared among individuals across the globe^3^. In addition, no significant genetic differentiation among Malaysian populations of *P. flavescens* was found, despite a high level of gene flow determined by mismatch distribution and neutrality tests, which also provided evidence of demographic expansion during the Pleistocene (190,000–260,000 years ago)^11^.

A less-mobile species may be expected to show some evidence of haplotype clustering according to geographic region. A portion of mitochondrial *CO1* gene sequence analysis of the tiny dragonfly *Nannophya pygmaea* showed overall low genetic diversity among 68 *N. pygmaea* individuals collected over six habitats in Korea^16^. Although these geographic populations of *N. pygmaea* in Korea clustered into two groups, genetic isolation by distance was not detected^16^.

Microsatellites are the most popular genetic marker owing to their high abundance, easily typed locus-specific codominance and Mendelian inheritance. The mitochondrial cytochrome *b* gene (*Cytb*) has often been used as a marker for studies of evolution and population structure and to resolve taxonomic conflicts in many animal groups^17^. In the present study, we used partial sequences of *Cytb* and microsatellite markers to analyze the population structure, genetic divergence and demographic history among individuals of *P. flavescens* collected during an intensive sampling in the eastern monsoon region of China. Such investigations can improve our knowledge of the migratory behavior of *P. flavescens* in China and may also contribute to investigations of the evolution of migration. Additionally, the sampling and analytical methods used here may provide a potential model to study the migration of other insects.

## Results

### Mitochondrial DNA analysis

From 583 individuals of *P. flavescens*, 542 sequences including 477 bases in the mtDNA *Cytb* genes were obtained. Of the total genetic characters, 430 bases were conserved, and 47 polymorphic sites were found in the alignment of 542 *Cytb* sequences, with 12 singleton polymorphic sites (20%) and 35 parsimony informative sites (80%). Genetic diversity and the distribution of haplotypes among different populations of *P. flavescens* based on the *Cytb* sequences are shown in Table 1. The number of haplotypes (NH) in each population ranged from 7 to 17 (mean 12). CS (See Supplementary Table 1 for code definitions and locations of each population) had the most haplotypes (NH = 17) and PL had the fewest haplotypes (NH = 7). Among the 77 haplotypes, haplotype H6 was the most frequent and widely distributed, being shared by 206 samples among 19 populations. The second-most frequent haplotype was H1, shared by 145 individuals among 19 populations. Thus, H6 and H1 were the primary haplotypes and were shared by individuals from different populations. The global haplotype diversity (HD) ranged from 0.662 (population PL) to 0.926 (HZ) (mean = 0.810). Nucleotide diversity (π) ranged from 0.003 (PL) to 0.009 (CS and QF) (mean = 0.006). The number of transitions was 311 and transversions numbered 48. Based on the combined populations, all 19 populations had high haplotype diversity and low nucleotide diversity (Table 1). These haplotypes are clustered into three branches but have no obvious geographical distribution in a median-joining network (Supplementary Fig. 1).

**Table 1:** Distribution of haplotypes and molecular diversity based on *Cytb* sequence from 542 individuals from among 19 populations of *Pantala flavescens* in China.

Pop	NS	NH	HD	π	TS	TV	Tajima’s *D*	*P*	Fu’s *F*_S_	*P*	*S*
Zhengzhou	27	9	0.732	0.005	311	48	−1.90	0.014	−1.505	0.241	21
Penglai	31	7	0.662	0.003	18	3	−1.518	0.045	−1.427	0.201	11
Hefei	33	11	0.786	0.005	11	0	−1.415	0.066	−2.968	0.06	17
Wuhan	35	11	0.748	0.006	14	3	−1.358	0.062	−2.212	0.169	19
Panjin	32	12	0.849	0.006	16	3	−0.738	0.268	−3.517	0.041	14
Haerbin	30	12	0.756	0.005	13	1	−1.976	0.006	−4.631	**0.011**	21
Guiyang	31	10	0.785	0.008	15	4	−1.085	0.133	−0.671	0.415	21
Guangzhou	28	10	0.672	0.004	18	3	−1.840	0.02	−3.569	0.02	16
Guilin	31	13	0.869	0.007	18	3	−0.940	0.176	−3.034	0.104	20
Langfang	29	11	0.837	0.005	15	1	−1.406	0.061	−3.122	0.062	17
Changsha	31	17	0.903	0.009	16	4	−1.113	0.119	−6.693	**0.004**	24
Changchun	31	13	0.804	0.006	16	1	−1.582	0.046	−4.6844	**0.015**	20
Qianfei	28	15	0.862	0.009	19	5	−0.898	0.177	−5.083	**0.018**	21
Jiangxi	29	13	0.8479	0.006	21	5	−1.297	0.086	−5.050	**0.005**	17
Ninxia	35	15	0.783	0.006	17	4	−1.441	0.068	−6.736	**0.002**	19
Chifeng	22	9	0.853	0.006	18	3	−0.251	0.456	−1.403	0.244	12
Hanzhong	17	11	0.926	0.007	16	1	−1.034	0.177	−4.102	**0.015**	16
Xinxiang	11	8	0.89	0.004	20	3	−1.809	0.023	−3.894	0.03	10
Taiyuan	33	12	0.782	0.005	19	0	−1.251	0.091	−4.551	**0.002**	16

NS: number of *Cytb* sequences; NH: number of haplotypes; HD: haplotype diversity; π: nucleotide diversity; TS: no. of transitions; TV: no. of transversions; S: number of polymorphic sites; *P* > 0.02 means no significant difference.

### Population genetic structure and Bayesian clustering based on mitochondrial data

Pairwise *F*_st_ values ranged from −0.003 to 0.090, with the highest differentiation observed between CC and LF and the lowest between HEB and QF (Supplementary Table S2). There was an intermediate level of differentiation between 66 populations (0.05 < *F*_st_ < 0.15), and the other 105 populations with no differentiation (*F*_st_ < 0.05). The *F*_st_ matrix based on mitochondrial genes showed that 133 of the 171 population pairs had no significant differentiation (Supplementary Table S2).

The AMOVA of the mtDNA data revealed 1.91% of the genetic variation was among population within groups, whereas the remaining (98.07%) came from variation within the populations. Results of the AMOVA test based on mtDNA markers in different populations of *P. flavescens* are shown in Table 2.

**Table 2:** Results of analysis of molecular variance (AMOVA) test on microsatellite and *Cytb* markers in different populations of *Pantala flavescens* in China.

Marker	Source of variation	Sum of squares	Variance components	Percentage variation	*S*tatistic
Microsatellite	Hierarchical AMOVA (*K* = 3)				
	Among groups	4.85	0.00285 V_a_	0.13	*F*_CT_: 0.00133
	Among populations within groups	43.834	0.08961 V_b_	4.18	*F*_SC_: 0.04189***
	Within populations	2182.84	2.04962 V_c_	95.68	*F*_ST_: 0.04316***
*Cytb*	Hierarchical AMOVA (*K = *3)				
	Among groups	4.85	0.00038 V_a_	0.02	*F*_CT_: −0.008
	Among populations within groups	43.834	0.02887 V_b_	1.91	*F*_SC_: 0.0197***
	Within populations	903.383	1.48583 V_c_	98.07	*F*_ST_: 0.0190***

***P < 0.001.

**Figure 1:**
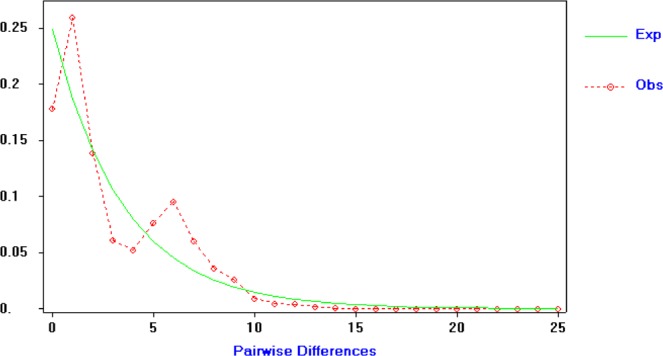
Frequencies of the observed and expected pairwise differences (the mismatch distribution) in the samples of *Pantala flavescens* from 19 populations in China.

When the 19 populations were regarded as a whole, Tajima’s *D* and and Fu’s *F*_S_ statistic are statistically negative but not significant (*P* > 0.02) (Table 1). At the same time, the bimodal mismatch distribution (Fig. 1) and three phyletic clusters of the mtDNA haplotype network also indicated no demographic expansion (Supplementary Fig. S1). For most populations in the eastern monsoon regions of China, Tajima’s *D* and Fu’s *F*_S_ was negative (Table 1), but not significant (*P* > 0.02). The results showed that these populations were in a stable state, with no recent bottleneck or a rapid population expansion. However, for the other populations (Table 1), Tajima’s *D* and Fu’s *F*_S_ statistic values are negative with *P* values being significant (*P* < 0.02), which showed these populations had experienced a recent population expansion.

### Microsatellite analysis

In a microsatellite analysis of 542 DNA samples from 19 populations using 10 microsatellite markers, Fisher’s test indicated 142 of 190 locus–population combinations deviated significantly from Hardy–Weinberg Equilibrium (HWE). According to a Micro-checker analysis, the null allele was very low, mostly less than 0.01 among 10 microsatellite markers; therefore, the presence of null alleles had almost no influence on the analysis of *F*_st_. The genetic parameters of these populations based on the 10 microsatellite markers among the 19 populations are summarized in Table 3. For 10 microsatellite markers across all populations, allelic richness (*A*_r_) ranged from 5.33 in population ZZ to 6.95 in CF (mean 5.94); 140 alleles were obtained, with 6 alleles (*N*a) across microsatellite loci in XX to 8.9 in HZ (mean 7.35). The mean effective number of alleles (*N*e) across microsatellite markers was 3.06, ranging from 3.03 in HEB to 4.41 in GL. The mean value of Shannon’s index (*I*) across microsatellite markers was 1.46, ranged from 1.34 (GZ) to 1.66 in (HZ). The observed heterozygosity (*H*_o_) ranged from 0.36 in QF to 0.55 in JX (mean 0.48) and expected heterozygosity (*H*_e_) ranged from 0.64 in HEB to 0.75 in HZ (mean 0.68). The unbiased expected heterozygosity (u*H*_E_) ranged from 0.65 in HEB to 0.77 in CF (mean 0.69). Overall, there was a high level of genetic diversity for all microsatellite loci in the study regions.

**Table 3:** Genetic diversity indices of 10 microsate markers in 19 populations of *Pantala flavescens* in China (See Table S1 for code definitions and locations of each population).

Pop.	*N*	*N*a	*N*e	*I*	*H*_o_	*H*_e_	u*H*_e_	*A*_r_
CC	29	7.2	3.5335	1.4314	0.4332	0.6795	0.6917	5.675
HE	30	7.5	3.0289	1.3625	0.4421	0.6394	0.6503	5.738
CS	26	7.1	3.053	1.3626	0.4344	0.6615	0.6749	5.799
QF	29	7.4	3.1712	1.3585	0.3562	0.6466	0.658	5.592
JX	26	7.6	3.4192	1.4699	0.549	0.6892	0.7027	5.908
Pj	28	7.5	3.2453	1.3986	0.3762	0.6569	0.669	5.946
HF	28	7.1	3.1876	1.3469	0.4848	0.6424	0.6541	5.510
GY	29	7.2	3.9612	1.5069	0.5416	0.7036	0.7161	6.075
GL	28	7.2	4.4138	1.5517	0.4726	0.7252	0.7384	6.271
NX	30	7.5	3.4187	1.4349	0.4955	0.6663	0.6776	5.905
TY	30	7.1	3.5205	1.4742	0.4892	0.6978	0.7099	5.671
ZZ	29	6.6	3.58	1.4221	0.4713	0.6908	0.7032	5.334
WH	31	8.5	3.9298	1.5534	0.5452	0.7155	0.7272	6.271
PL	30	7.3	3.9862	1.5442	0.5378	0.7272	0.7398	6.104
GZ	28	6.8	3.0807	1.3412	0.5089	0.6447	0.6565	5.553
LF	29	7.9	3.5485	1.4894	0.4959	0.6829	0.6949	6.254
CF	22	7.3	4.3012	1.6197	0.5255	0.7486	0.7662	6.951
HZ	26.	8.9	4.2964	1.6616	0.4619	0.7489	0.7636	6.480
XX	15	6	3.743	1.4352	0.4193	0.6962	0.7211	5.729
Mean	28	7.353	3.60	1.461	0.476	0.688	0.70	5.936

*N*, number of samples; *N*a, number of alleles; *N*e, number of effective alleles; *I*, Shannon’s index; *H*_e_, expected heterozygosity; u*H*_e_, unbiased expected heterozygosity; *H*_o_, observed heterozygosity; *A*_r_, allelic richness.

### Population genetic structure and Bayesian clustering based on microsatellite data

**Figure 2:**
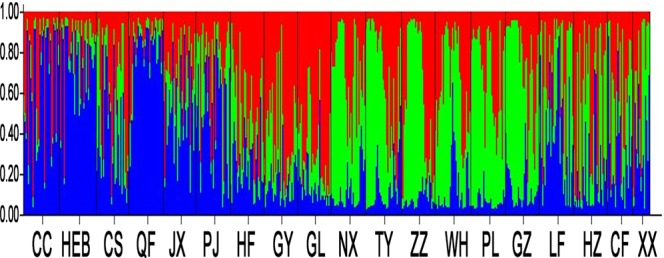
Bayesian clustering analysis of 19 populations of *P. flavescens*. Population codes are given in Table S1. Each individual is represented by a vertical bar displaying membership coefficients for each genetic cluster. Blue, green and red represent the three clades.

The *F*_st_ matrix based on microsatellite data showed that 101 of the 171 population pairs had significant differentiation (Supplementary Table S3). Pairwise *F*_st_ values based on microsatellite data ranged from 0.001 to 0.09, with the highest differentiation between LF and CC and the lowest between GY and GL (Supplementary Table S3). There was intermediate differentiation between 65 population pairs (0.05 < *F*_st_ < 0.15), and the other 106 population pairs had no differentiation (*F*_st_ < 0.05). The Bayesian clustering analysis revealed the presence of three distinct clusters, and each population was a mixture including individuals from three clusters (Supplementary Fig. 2, Fig. 2). Figure 2 revealed allelic similarities among these populations and showed differences in the frequencies of common alleles among them.

The AMOVA of the microsatellite data revealed 4.18% genetic variation among population within groups, whereas the remaining (95.68%) was genetic variation within populations.

A high distribution proportion was obtained by the partial Bayesian method in Geneclass, and 95.7% (519/542) of the individuals were assigned to the 19 populations. The remaining 23 individuals might be migrants from other areas to the sampling regions. GENECLASS identified that all migratory individuals were offspring of migrants and that none were first-generation migrants.

### Mantel test for isolation by distance

Our observation showed no significant correlation between genetic distance and geographic distance of these populations based on the two types of markers (microsatellite genotypes: *Z* = 8299.1787, *r* = 0.0854, *P* = 0.804, and *Cytb*: *Z* = 2863.798, *r* = −0.1002, *P* = 0.147), which may be reflective of a lack of phylogenetic divergence among the individuals across the study areas. This result also suggested migratory dragonflies could colonize and exchange genes with local populations.

## Discussion

Our results showed high rates of gene flow occurred among 19 geographic populations. Natural dispersal ability and long-distance migration are the most important factors contributing to a higher level of gene flow and consequent slowing or limitation of geographic differentiation^11,18^. In our current study, *Pantala flavescens* had high haplotype interconnection among 19 populations in the eastern monsoon regions of China and a lack of phylogeographic structuring. Large-scale migrations of *P. flavescens* resulted in high rates of gene flow, lower genetic diversity. and the lack of physical barriers to gene flow.

The present study revealed deviations from Hardy–Weinberg equilibrium at 10 microsatellite markers in 19 populations, which was due to the low heterozygosity, which was further confirmed by the MICRO-CHECKER analysis. Ability to migrate long distances of dragonflies might be the major factors causing the deviation from Hardy–Weinberg equilibrium among the studied populations. This result is consistent with previous reports^19^. Wright (1978) suggested that if *F*_st_ = 0, then the two populations lack differentiation; when *F*_st_ = 0.05–0.15, the populations are moderately differentiated, and when *F*_ST_ = 0.15–0.25, the populations are highly differentiated^20^. In our experiment, different populations had low to moderate genetic differentiation based on *F*_st_ values: from −0.003 to 0.09 (Supplementary Table S2) and from 0.001 to 0.09 (Supplementary Table S3). For both the SSR and mtDNA molecular markers, the percentage of variation mainly existed within populations (>95%), whereas the percentage of variation among groups and among populations within groups clustered by 19 populations was less than 5% (Table 2). Compared with mitochondrial genes, microsatellite DNA might reveal more information on variation (Supplementary Tables S2 and S3), gene flow, bottlenecks and population divergence. *P. flavescens* in the eastern monsoon regions of China was classified into three genetic clusters, which was supported by the AMOVA result (Table 2) and STRUCTURE analysis (Supplementary Fig. 2, Fig. 2).

The median-joining network revealed a close relationship among haplotypes, suggesting that *P. flavescens* populations share a recent history without long-term genetic isolation. These ancestral haplotypes (H6 and H1) of mtDNA were widely distributed in all populations, and the haplotypes in 19 populations formed three clusters, but had no obvious geographic divisions, which indicates that geographic barriers and climatic factors have little influence on migration of this dragonfly in different regions.

Estimates of *Nm* are often taken at face value as the approximate number of migrants moving among populations^21^. In our experiment, different populations had a high number of individuals dispersed (*Nm* = 4.326), thus avoiding the genetic differentiation that arises from genetic drift and explaining the low inter-subpopulation genetic variation. Our results showed a high rate of gene flow and lack of population differentiation among 19 studied populations. At the same time, genetic differentiation and geographic distance were not correlated, so isolation by distance does not appear to be a barrier for gene flow.

A less-mobile species may be expected to harbor some evidence of haplotype clustering according to geographic region. *Nannophya pygmaea* is very small and unlikely to migrate over large-scale regions, which likely contributes to its overall low diversity and genetic isolation by distance^16^. The endangered damselfly *Coenagrion mercuriale* (Charpentier, 1840) is a weak flier^22^, and significant genetic differentiation between sites can be prevented if sites are <2 km apart and not separated by a physical barrier. Nevertheless, dispersal by *C. mercuriale* is sufficiently restricted so that genetic structure can result from isolation by distance develops within 10 km^22^.

Our results suggested that *P. flavescens* could successfully colonize and adapt new habitats. They were able to disperse randomly and exchange genes with local populations which lead to high rates of gene flow among 19 geographic populations. However, the migration of *P. flavescens* on a global scale and the potential ecological impacts of their migratory behavior remain unknown and need further research.

## Materials and Methods

### Insect materials

**Figure 3:**
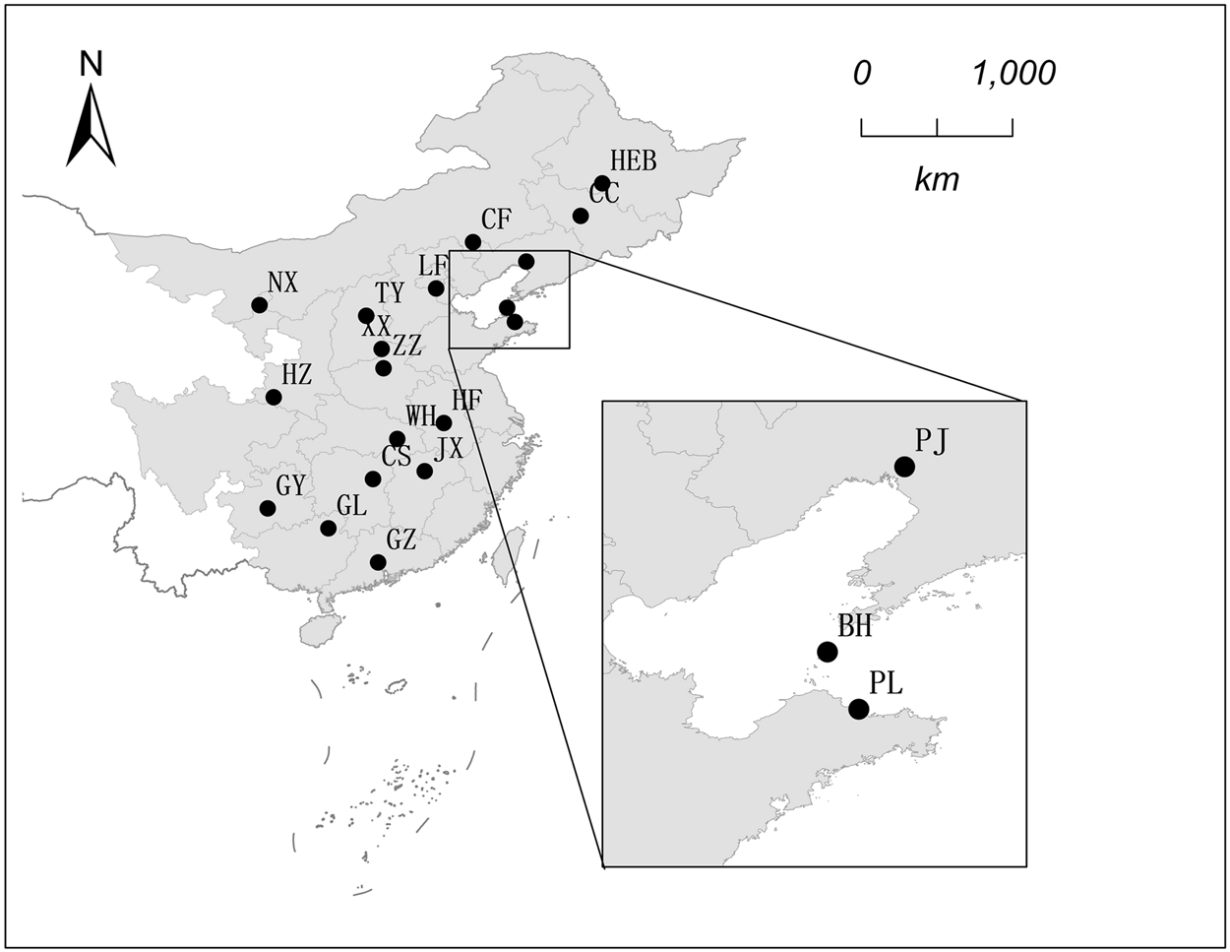
Locations of 19 sampling sites of *Pantala flavescens* in eastern the monsoon region of China during 2013 and 2014. Population codes are given in Table S1.

In total, 583 individuals of *P. flavescens* were sampled from 19 geographic sites in 18 provinces in China, from June 2013 through October 2014 (Fig. 3; Supplementary Table S1). These samples were collected through a sweep net and stored at −20 °C.

### DNA extraction

Genomic DNA was extracted from the thorax of individual adults using the TIANamp Genomic DNA Kit (Tiangen Biotech, Beijing), and preserved at −20 °C in the refrigerator.

### Mitochondrial DNA sequencing and analysis

Partial regions of the mitochondrial gene *Cytb* were amplified using published primers^23^. Each PCR amplification was performed in 30 μL reactions with each reaction consisting of 15 μL of 2× Taq Master Mix solution (CoWin Biotech Co., Beijing), 1 μL of DNA template, 1 μL of each primer (forward and reverse, both diluted 10×), and 12 μL of RNase-free water. The PCR reactions were performed in a Techne thermocycler (Germany) with the following program: 5 min initial denaturation step at 94 °C; 35 cycles of 45 s at 94 °C, 45 s at 55 °C, 1 min at 72 °C; and a final extension 5 min at 72 °C. Amplification products of *Cytb* were sequenced on an ABI 3730 DNA Sequencer (Applied Biosystems, USA). Mitochondrial DNA sequences were manually checked and aligned with ClustalX 1.85^24^, using the multiple alignment default parameters. Nucleotide composition, parsimony informative sites, variable sites and conserved sites were calculated with MEGA 6^25^. Molecular diversity indices such as nucleotide diversity and haplotype diversity were analyzed in DnaSP 4.0^26^. Genetic differentiation (fixation index *F*_st_) between populations was calculated using mtDNA data and Arlequin 3.0 with 10000 permutations^27^. Analysis of molecular variation (AMOVA) based on mtDNA data, as implemented in Arlequin 3.0^27^, was used to test for hierarchical genetic structure of the populations.

Network 2.0^28^ software was used to construct a median-joining network. Demographic history changes were analyzed for *P. flavescens* using two neutrality tests, Tajima’s *D* (1989)^29^ and Fu’s *F*s (1997)^30^, which explained a recent population bottleneck or population expansion. Mismatch distributions count the number of site differences between each pair of sequence in a sample and use the calculation result to build a histogram. According to the coalescent theory, a population usually shows a unimodal mismatch distribution following a population demographic expansion^31^.

### Microsatellite genotyping and analysis

Total DNA was extracted from 583 individuals for PCR and genotyping for 10 microsatellite loci developed for *P. flavescens*^19^. MICRO-CHECKER 2.2.3 was used to detect genotyping errors in microsatellite data due to null alleles, stuttering, or allele dropout using 1000 randomizations^32^. Deviations from Hardy–Weinberg equilibrium (HWE) in all the loci of each population and linkage disequilibrium between pairs of loci were assessed using Genepop 3.4^33^. To study the genetic diversity of different geographic populations, we used GenAlEx 6.41^34^ to calculate mean number of alleles (*N*_a_), effective number of alleles (*N*_e_), Shannon’s index (*I*); expected and observed heterozygosity (*H*_e_ and *H*_o_) and unbiased expected heterozygosity (u*H*_E_). Allelic richness (*A*_r_), fixation index (*F*_st_), and inbreeding coefficient (*F*_is_) among these sites were analyzed in FSTAT 2.9.3^35^. Genetic differentiation between all pairs of populations was calculated in Arlequin 3.0^27^.

Analysis of molecular variation (AMOVA) based on microsatellite data, as implemented in Arlequin 3.0^27^, was used to test for hierarchical genetic structure of the populations. We used STRUCTURE 2.3.3^36^ and its nonspatial algorithm to further assess the degree of population differentiation within and between the 19 populations based on microsatellite data. The allelic frequencies for different populations and the admixture model were used to class different individuals into corresponding population clusters. Simulation was run 7 times for each value of *k* for 10^6^ iterations after a burn-in period of 30,000. To determine the optimal number of groups (*K*), we utilized both the log likelihood [ln Pr (*X*/*K*)] method as recommended by Pritchard *et al*.^37^ and the Δ*K* statistic of Evanno *et al*.^36^. Individuals of the first migrant generation for each population were detected using the L-home likelihood computation in GENECLASS 2^38^.

### Mantel test for isolation by distance

Correlation tests were conducted between the geographic distance and the corresponding genetic differentiation matrix of these populations in the Mantel test. The geographical and genetic distances should be positively correlated if the dispersal of *P. flavescens* is influenced by distance. To determine whether movement patterns were limited by spatial scale, we ran isolation by distance analysis between pairwise linearized genetic and log-geographic distance data using a Mantel test in IBDWS 3.23^39^.

## Supplementary information

Supplement material
