# Supplementary material for: Genetic Diversity and Demographic History of Globe Skimmers (Odonata: Libellulidae) in China Based on Microsatellite and Mitochondrial DNA Markers
Source: Sci Rep. 2019 Jun 13;9:8619. doi: 10.1038/s41598-019-45123-0 (PMC6565731; doi:10.1038/s41598-019-45123-0)
Supplement: Supplementary file 1 — Supplement material [file 41598_2019_45123_MOESM1_ESM.pdf]

Supplements

Genetic Diversity and Demographic History of Globe Skimmers (Odonata: Libellulidae) in China Based on Microsatellite and Mitochondrial DNA Markers

Ling-zhen Cao[1,2] and Kong-ming Wu[1*]

1 Institute of Plant Protection, Chinese Academy of Agricultural Sciences, Beijing, 100193, P.R. China

2. College of Life Science, Jiangxi Normal University, Nanchang, Jiangxi, 330022 P.R. China

[*] Corresponding author: Kongming Wu; Email: kmwu@ippcaas.cn; Tel: +86(0)1082105551

Mailing address: Institute of Plant Protection, Chinese Academy of Agricultural Sciences, Beijing 100193, P.R. China.

Table S1 Collection information for samples of *Pantala flavescens* used in this study.

Location	Code	Coordinates	Nbs	Location	Code	Coordinates	Nbs
Langfang	LF	39.5379 116.7104	32	Guilin	GL	25.2618 110.3009	31
Taiyuan	TY	37.9055 112.5474	36	Guiyang	GY	26.4411 106.687	34
Chifeng	CF	42.2922 118.9087	25	Ningxia	NX	38.5415 106.2035	30
Penglai	PL	37.5427 121.4001	33	Hanzhong	HZ	33.0557 107.0481	16
Hefei	HF	31.5237 117.17	32	Panjin	PJ	41.1324 122.0792	35
Zhengzhou	ZZ	34.7845 113.5837	33	Changchun	CC	43.8538 125.3127	35
Xinxiang	XX	35.9314 113.4736	15	Haerbin	HEB	45.787 126.5942	35
Nanchang	JX	28.6687 116.031	35	Guangdong	Gz	23.2238 113.2543	30
Changsha	CS	28.1925 112.9579	32	Qianfei	QF	37.9289 120.7479	32
Wuhan	WH	30.5811 114.4015	32	Total			583

Nbs, number of samples

Table S2 Pairwise F_{ST} values among 19 populations (Pop.) of *Pantala flavescens* in China based on*Cytb* data.

Pop	XX	CF	HZ	LF	GZ	PL	WH	ZZ	TY	NX	GL	GY	HF	PJ	JX	QF	CS	HEB	CC
XX	0.000																		
CF	0.026	0.000																	
HZ	0.028	0.004	0.000																
LF	0.014	0.028	0.026	0.000															
GZ	0.064	0.049	0.067	0.078	0.000														
PL	0.070	0.029	0.040	0.076	0.021	0.000													
WH	0.061	0.036	0.045	0.073	0.006	0.002	0.000												
ZZ	0.003	0.032	0.020	0.007	0.068	0.070	0.062	0.000											
TY	0.024	0.011	0.021	0.016	0.052	0.046	0.054	0.018	0.000										
NX	0.011	0.037	0.034	0.010	0.040	0.053	0.041	0.008	0.019	0.000									
GL	0.038	0.005	0.008	0.035	0.075	0.045	0.047	0.036	0.023	0.043	0.000								
GY	0.029	0.009	0.008	0.021	0.061	0.045	0.038	0.019	0.020	0.016	-0.001	0.000							
HF	0.025	0.017	0.024	0.008	0.066	0.060	0.062	0.018	0.011	0.030	0.016	0.015	0.000						
PJ	0.044	0.041	0.050	0.009	0.075	0.077	0.080	0.027	0.022	0.027	0.054	0.038	0.021	0.000					
JX	0.033	0.012	0.024	0.019	0.050	0.044	0.047	0.024	0.009	0.028	0.024	0.017	0.013	-0.002	0.000				
QF	0.053	0.058	0.071	0.071	0.032	0.062	0.052	0.073	0.069	0.049	0.070	0.070	0.068	0.082	0.070	0.000			
CS	0.052	0.035	0.043	0.038	0.062	0.061	0.059	0.043	0.031	0.046	0.046	0.037	0.033	0.023	-0.002	0.073	0.000		
HEB	0.056	0.057	0.075	0.077	0.018	0.064	0.049	0.074	0.068	0.048	0.075	0.069	0.072	0.075	0.067	-0.003	0.079	0.000	
CC	0.065	0.030	0.053	0.090	0.038	0.046	0.036	0.077	0.066	0.063	0.046	0.042	0.067	0.085	0.052	0.036	0.061	0.027	0.000

Significance level= 0.0100; red means $p > 0.01$.

Table S3 Pairwise F_{st} values among 19 populations (Pop.) of *Pantala flavescens* in China based on 10 microsatellite loci.

Pop	CC	HEB	CS	QF	JX	PJ	HF	GY	GL	NX	TY	ZZ	WH	PL	GZ	LF	HZ	CF	XX
CC	0.000																		
HEB	0.027	0.000																	
CS	0.061	0.079	0.000																
QF	0.036	0.003	0.073	0.000															
JX	0.052	0.067	0.002	0.070	0.000														
PJ	0.085	0.075	0.023	0.082	0.002	0.000													
HF	0.067	0.072	0.033	0.068	0.013	0.021	0.000												
GY	0.042	0.069	0.037	0.070	0.017	0.038	0.015	0.000											
GL	0.046	0.075	0.046	0.070	0.024	0.054	0.016	0.001	0.000										
NX	0.063	0.048	0.046	0.049	0.028	0.027	0.030	0.016	0.043	0.000									
TY	0.066	0.068	0.031	0.069	0.009	0.022	0.011	0.020	0.023	0.019	0.000								
ZZ	0.077	0.074	0.043	0.073	0.024	0.027	0.018	0.019	0.036	0.008	0.018	0.000							
WH	0.036	0.049	0.059	0.052	0.047	0.080	0.062	0.038	0.047	0.041	0.054	0.062	0.000						
PL	0.046	0.064	0.061	0.062	0.044	0.077	0.060	0.045	0.045	0.053	0.046	0.070	0.002	0.000					
GZ	0.038	0.018	0.062	0.032	0.050	0.075	0.066	0.061	0.075	0.040	0.052	0.068	0.006	0.021	0.000				
LF	0.090	0.077	0.038	0.071	0.019	0.009	0.008	0.021	0.035	0.010	0.016	0.007	0.073	0.076	0.078	0.000			
HZ	0.053	0.075	0.043	0.071	0.024	0.050	0.024	0.008	0.008	0.034	0.021	0.020	0.045	0.040	0.067	0.026	0.000		
CF	0.030	0.057	0.035	0.058	0.012	0.041	0.017	0.009	0.005	0.037	0.011	0.032	0.036	0.029	0.049	0.028	0.004	0.000	
XX	0.065	0.056	0.052	0.053	0.033	0.044	0.025	0.029	0.038	0.011	0.024	0.003	0.061	0.070	0.064	0.014	0.028	0.026	0.000

Significance level = 0.0100; red means $p > 0.01$.

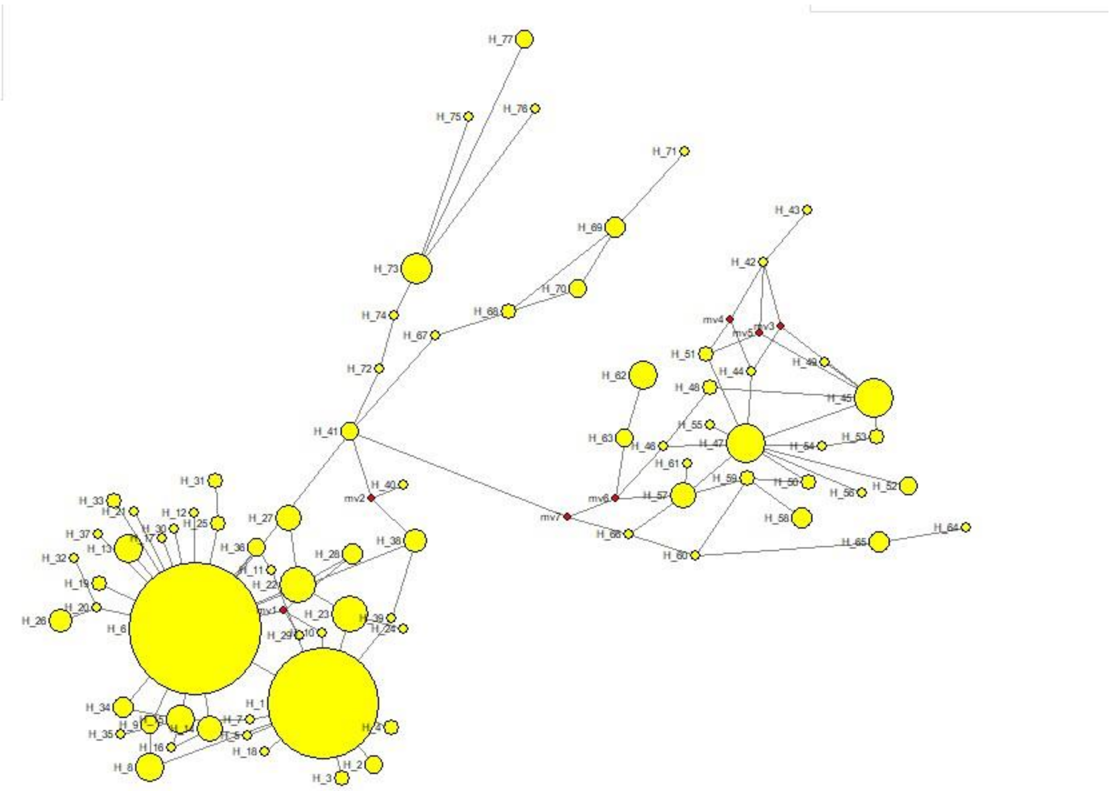

Figure S1 Median-joining network of haplotypes based on mitochondrial *Cytb* sequences

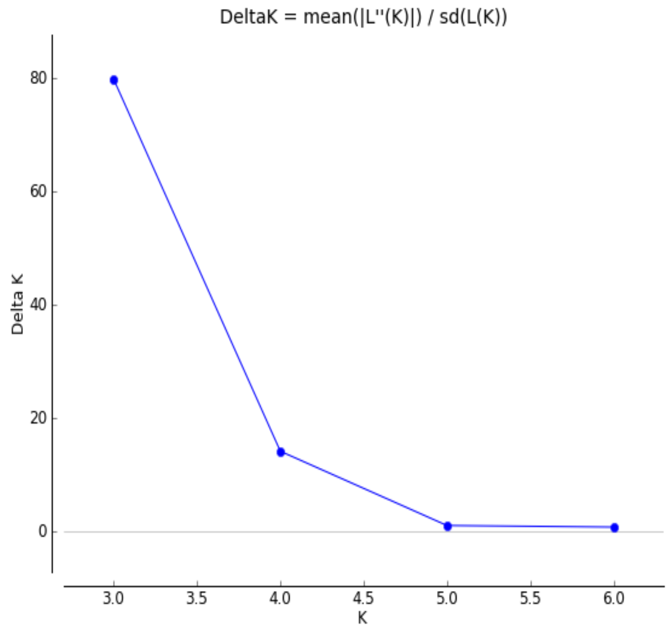

Genotype clusters (*k*)

Figure S2 Inference of the number of genetic clusters (*K*) from STRUCTURE simulations for *Panala flavescens*.
